# The Risk of Gastrointestinal Hemorrhage in Low-Dose Aspirin Users with Diabetes Mellitus: Systematic Review and Meta-Analysis

**DOI:** 10.1155/2020/9824615

**Published:** 2020-08-03

**Authors:** Yashuo Wang, Wei Wang, Bin Wang, Yunyang Wang

**Affiliations:** ^1^College of Life Sciences, Qingdao University, China; ^2^Institute of Oceanology, Chinese Academy of Sciences, China; ^3^Department of Medical Microbiology, Qingdao University Medical College, China; ^4^Department of Endocrinology and Metabolism, The Affiliated Hospital of Qingdao University, China

## Abstract

**Background:**

Our aim was to assess the risk of gastrointestinal (GI) hemorrhage associated with diabetes among patients taking low-dose aspirin (≤325 mg/day).

**Methods:**

A systematic search was conducted for publication in English and Chinese using term equivalents for “GI hemorrhage”, “aspirin”, and “diabetes mellitus” up till April 2020. Electronic databases include PUBMED, EMBASE, Cochrane Library databases, Chinese National Knowledge Infrastructure (CNKI), Wanfang Database, and VIP Database. Two independent authors searched databases and reviewed abstracts for comprehensive studies keeping adequate study quality. Data of weighted odds ratios were statistically evaluated and potential bias was checked.

**Results:**

Among 446 publications, eight case-control researches, including 1601 patients, were deemed for this meta-analysis. Patients with diabetes were associated with a higher risk of GI hemorrhage than patients without diabetes: the summary ORs were 3.10 (95% CI, 2.35–4.09). The heterogeneity of the reports was not significant (Chi^2^ = 3.39, *P* = 0.85; *I*^2^ = 0%).

**Conclusion:**

The meta-analysis showed that aspirin users with diabetes were more likely to have GI hemorrhage. Hence, when treating diabetics with aspirin, the increased risk of GI bleeding should be taken in consideration.

## 1. Introduction

As one of the most widely used agents among the world, aspirin is commonly used for treatment to migraine, pain, fever, or colds, and also for the prevention of cardio- and neurovascular diseases [1, 2]. The American Heart Association (AHA), the American College of Cardiology Foundation (ACCF), and the European Society of Cardiology (ESC) recommend the use of aspirin in all patients with coronary artery disease [3–5]. The definition of “low-dose aspirin” is based on the North American formulation of single analgesic-strength tablets [6]. Most clinicians recommend a 100 mg tablet or less as the maximum daily dosage for treatment.

Nonetheless, long-term therapy with aspirin is reported to carry a risk of gastrointestinal (GI) adverse effects, including ulceration and bleeding [7–9]. Derry's research of a meta-analysis suggests that GI hemorrhage occurred in 2.47% of patients with long-term use of aspirin compared with 1.42% taking placebo (odds ratio 1.68; 95% CI, 1.51-1.88). And no evidence indicates that reducing the dose or using modified release formulations would reduce the risk of GI hemorrhage [10]. Aspirin might cause GI bleeding via inhibition of platelet aggregation and systemic effects on epithelial and endothelial cells of mucosa, therefore results in a lower rate of cell proliferation and migration [11].

Whether diabetes mellitus (DM) is an independent risk factor for GI bleeding among aspirin users is conflicting. A cohort study revealed that DM was an independent risk factor for upper GI bleeding among aspirin users [12]. However, another cohort research based on 186 425 individuals suggested that the use of aspirin was associated with a greater risk of major bleeding in most of the subgroups investigated but not in individuals with DM (IRR, 1.09; 95% CI, 0.97-1.22) [13]. Therefore, in this study, we conducted an updated systematic review with the aim of identifying articles suitable for meta-analysis which reported GI hemorrhage in aspirin users with or without DM.

## 2. Methods

This article was conducted by the guidance of the PRISMA statement.

### 2.1. Search Strategy

Publications in English were searched in PUBMED, EMBASE, and the Cochrane Library database, and reports in Chinese were searched in Chinese National Knowledge Infrastructure (CNKI), Wanfang database, and VIP database up to April 2020. We searched the term equivalents for “GI hemorrhage”, “aspirin”, and “diabetes mellitus”. Detailed search strategies could be found in the Supplementary Material (available here). Two authors performed searching separately. Relevant articles were identified through the combination of electronic searching and manual checking of references from related publications.

### 2.2. Selection Criteria

Publications used in this meta-analysis were original case-control studies, which reported GI hemorrhage happening among aspirin users who have DM or not. Endoscopic documentation of GI bleeding was preferred but not essential. The inclusion criteria were the following: (1) case-control studies; (2) patients taking low-dose aspirin (≤325 mg/day); (3) outcomes including GI hemorrhage and/or peptic ulcer. According to the exclusion criteria, nonhuman researches, pharmacological experiments, single case reports, meta-analysis, reviews, guidelines, studies using concomitant drugs, and articles without full papers were foreclosed.

### 2.3. Appraisal of Study Quality and Data Extraction

The quality of studies included in this meta-analysis was assessed by two authors independently with the Newcastle-Ottawa Scale (NOS) [14]. Discrepancies in individual scores were discussed, and the mean score was calculated as the final one. Studies scoring seven or more were deemed as high quality.

Data were collected on baseline patient characteristics, aspirin dosage and duration, ratio of gender, and case numbers. All outcome data were extracted by two investigators (YS and YY).

### 2.4. Statistical Analysis

Meta-analysis summary statistics and heterogeneity were generated in the Fixed Effect model by the Mantel-Haenszel method with Review Manager 5.3. We calculated odds ratios and 95% confidence intervals (95% CIs) for GI bleeding. Heterogeneity was estimated as Chi^2^ and *I*^2^.

## 3. Results

### 3.1. Publications and Study Characteristics

**Figure 1:**
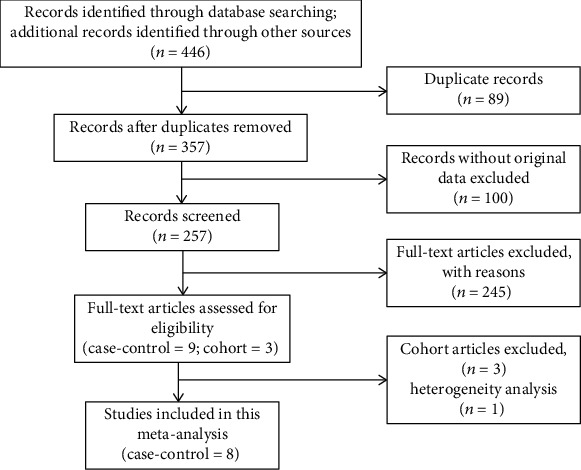
Flow chart of study selection.

In total, 343 English records and 104 Chinese records were initially retrieved. After excluding 89 duplicates, 357 articles were screened according to the inclusion criteria. One hundred nonclinical studies were excluded (e.g., single case reports, meta-analysis, reviews, guidelines or pharmacological experiments, nonhuman researches). Then, those not meeting our treatments and outcomes were foreclosed. Nine papers were checked and reviewed in detail, and one of them was excluded due to its heterogeneity. In summary, eight case-control studies, which fulfilled our inclusion criteria were identified for our meta-analysis. The flowchart of the selection process is provided in Figure 1.

The eight publications in this meta-analysis included 1601 patients in total. 1176 took low-dose aspirin while the dosages of the other 425 were not reported. Two of the eight studies did not mention whether endoscopically confirmation was performed, but the context made it highly possible. And the other six publications suggested endoscopic evidence of GI hemorrhage. The characteristics of these studies are listed in Table 1.

### 3.2. Quality Assessment

All of the eight papers reached five points by NOS, and four of the eight researches were high quality (scored as seven or more). The main reasons for studies rating lower than seven were the absence of community-based controls. The NOS scores were listed in Table 1.

### 3.3. Meta-Analysis

**Figure 2:**
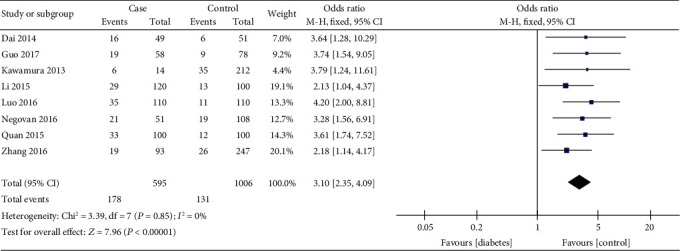
Forest plot of researches comparing GI hemorrhage in aspirin users with or without DM. The summary odds ratio and its 95% confidence interval were conducted in a Fixed Effect model by the Mantel-Haenszel method. Case, gastrointestinal bleeding patients. Control, no gastrointestinal bleeding patients. Events, diabetics.

**Figure 3:**
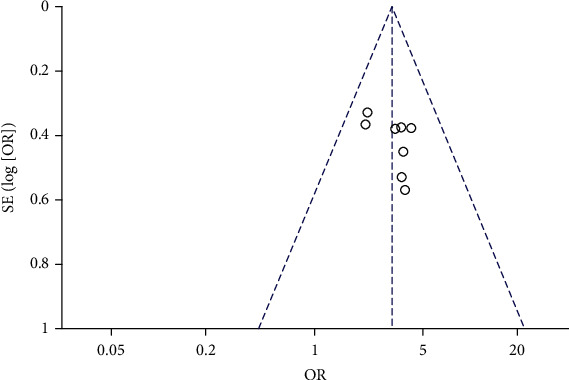
Funnel plot of heterogeneity of studies.

Raw data were extracted from these eight papers to generate ORs for GI hemorrhage in patients taking aspirin with or without DM (Figure 2). The summary OR was 3.10 (95% CI, 2.35-4.09). The pooled effected size in the meta-analysis indicated that GI hemorrhage was higher in aspirin users with DM than those without. In Figures 2 and 3, the heterogeneity of the reports was not significant (Chi^2^ = 3.39, *P* = 0.85; *I*^2^ = 0%).

### 3.4. Sensitivity Analysis

**Figure 4:**
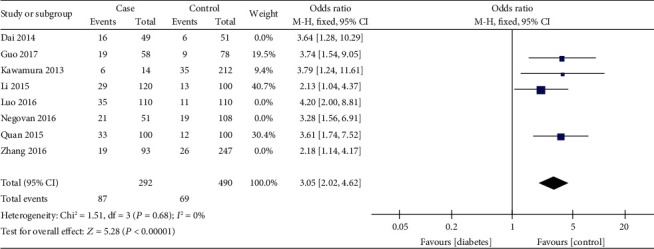
Forest plot of high-quality researches. Case, gastrointestinal bleeding patients. Control, no gastrointestinal bleeding patients. Events, diabetics.

We recalculated the pooled effected size for the analysis in which only high-quality studies were included. The summary OR was 3.05 with a 95% CI of 2.02–4.62 (Figure 4). The result was similar to which all studies were pooled.

## 4. Discussion

This meta-analysis suggests that DM is a risk factor for GI hemorrhage among patients who take low-dose aspirin. The OR values of all these eight studies included were more than two, and the summary OR was 3.1 (95% CI, 2.35–4.09). Sensitivity analysis of research quality yielded similar estimates of the increased frequency of GI bleeding associated with DM (OR, 3.05; 95% CI, 2.02–4.62). Our study offered a numerical value for consideration of aspirin treatment in patients with DM.

DM is considered to be associated with a hypercoagulable state [23]; however, a cohort study showed that DM increased GI bleeding risk even without taking aspirin [13]. So DM and aspirin might be two individual risk factors contributing to GI bleeding. DM may also enhance the mechanism by which aspirin causes GI bleeding. Aspirin induces prostaglandin depletion, damaging the gastric epithelial cell barrier [24]. The repairment of the cell barrier needs sufficient microcirculation, which may be interrupted in DM patients.

The total number of aspirin users is projected to rise, which poses substantial attention on the potential side effects such as GI bleeding. When treating patients with DM, the risk of GI bleeding should be taken in consideration. It is reasonable to suggest that patients receiving aspirin should be strongly considered for test-and-treat approach. Furthermore, an alternative medicine for aspirin may seem straightforward.

## 5. Conclusion

Despite the limitations, the consistency of our results (after sensitivity analysis) indicates that in patients taking low-dose aspirin, the likelihood of GI hemorrhage in individuals with DM is higher than those without DM. And further studies should help to elucidate whether the benefit of aspirin outweighs the risk in appropriate patients groups.

## 6. Strengths and Limitations

This research confirmed that DM is a risk factor for GI bleeding among aspirin users. The main strength is that it is the first meta-analysis focusing on case-control studies to investigate the contribution of DM to rates of GI hemorrhage in patients taking low-dose aspirin. Previous researches answered this question by performing random controlled trials. Limitations of our research lies in the quality of studies included. Because our data were derived from case-control studies, the comparability of groups can hardly be assured and unrecognized confounders could have influenced outcomes.

## Figures and Tables

**Table 1:** Characteristics of studies included in the meta-analysis.

Study	Ages	Gender: male : female	Aspirin dosage (mg/day)	Aspirin duration	Cases: inclusion criteria	NOS score
Dai et al. 2014 [15]	60.8 ± 9.6	64 : 40	100	3 months–15 years	Upper GI hemorrhage	5
Guo and Zhang 2017 [16]	61.0 ± 10.3	80 : 56	100	3 months–14 years	GI hemorrhage	7
Kawamura et al. 2013 [17]	NR	NR	75	≥3 months	Ulcer as a mucosal deficit >5 mm in diameter	8
Li et al. 2015 [18]	60.5 ± 12.7	118 : 102	100	≥3 months; mean months: 23.8 ± 17.8	Gastrorrhagia and/or duodenal hemorrhage	7
Luo 2016 [19]	57.0 ± 6.6	120 : 56	NR	≥2 months	Upper GI hemorrhage	5
Negovan et al. 2016 [20]	NR	NR	75-325	≥1 months	Gastroduodenal ulcer and/or hemorrhage	6
Quan 2015 [21]	64 ± 11.8	113 : 87	NR	≥2 months	Upper GI hemorrhage	8
Zhang et al. 2016 [22]	65.7 ± 16.2	210 : 130	≤100	≥7 days	GI hemorrhage	5

NR: not report; GI: gastrointestinal.

## Data Availability

The data in this meta-analysis are from previously reported studies and datasets, which have been cited. The processed data are available in Table 1 of our manuscript.

## Supplementary Materials

Supplementary Materials Summary of literature search strategies.
