# Supplementary material for: The Risk of Gastrointestinal Hemorrhage in Low-Dose Aspirin Users with Diabetes Mellitus: Systematic Review and Meta-Analysis
Source: Gastroenterol Res Pract. 2020 Aug 3;2020:9824615. doi: 10.1155/2020/9824615 (PMC7422351; doi:10.1155/2020/9824615)
Supplement: Supplementary Materials — Summary of literature search strategies. [file 9824615.f1.pdf]

Supplementary material

Summary of literature search strategies

| Electronic Database | Search term used | Number of matching publication |
|---|---|---|
| PUBMED | ("aspirin"[MeSH Terms] OR "aspirin"[All Fields] OR "acetylsalicylic acid"[MeSH Terms] OR "acetylsalicylic acid "[All Fields]) AND ("diabetes"[MeSH Terms] OR "diabetes"[All Fields] OR "diabetes mellitus"[MeSH Terms] OR "diabetes mellitus"[All Fields]) AND ("gastrointestinal hemorrhage"[MeSH Terms] OR "gastrointestinal hemorrhage"[All Fields] OR "Peptic Ulcer"[MeSH Terms] OR "Peptic Ulcer "[All Fields] OR "Peptic Ulcer Hemorrhage"[All Fields] OR "Hematochezia"[MeSH Terms] OR "Hematochezia"[All Fields] OR "Peptic Ulcer bleed*"[All Fields] OR "gastrointestinal bleed*"[All Fields] OR "stomach Hemorrhage"[All Fields] OR "stomach bleed*"[All Fields] OR "small intestine Hemorrhage"[All Fields] OR "small intestine bleed*"[All Fields] OR "duodenum hemorrhage"[All Fields] OR "duodenum bleed*"[All Fields]) | 131 |
| EMBASE | ('aspirin':ab,ti OR 'acetylsalicylic acid':ab,ti) AND ('diabetes' OR 'diabetes mellitus') AND ('gi bleeding':ab,ti OR 'gastrointestinal hemorrhage':ab,ti OR 'peptic ulcer':ab,ti OR 'peptic ulcer hemorrhage':ab,ti OR 'hematochezia':ab,ti OR 'peptic ulcer bleed':ab,ti OR 'gastrointestinal bleed':ab,ti OR 'stomach hemorrhage':ab,ti OR 'stomach bleed':ab,ti OR 'small intestine hemorrhage':ab,ti OR 'small intestine bleed':ab,ti OR 'duodenum hemorrhage':ab,ti OR 'duodenum bleed':ab,ti) | 145 |
| Cochrane Library databases | ("aspirin" OR "LDA" OR "acetylsalicylic acid") and ("diabetes mellitus" OR “diabetes”) and ("gastrointestinal hemorrhage" OR "peptic ulcer" OR "peptic ulcer hemorrhage" OR "hematochezia" OR "peptic ulcer bleed" OR "gastrointestinal bleed" OR "gastrointestinal hemorrhage" OR "stomach hemorrhage" OR "small intestine hemorrhage" OR "small intestine bleed" OR "duodenum hemorrhage" OR "duodenum bleed" OR "stomach bleed") | 66 |
